# Comparison of ultrasound-guided oblique-axis versus short-axis approaches for right internal jugular vein cannulation in the left decubitus position: a prospective, randomized, single-center trial

**DOI:** 10.1186/s12871-026-04360-3

**Published:** 2026-10-19

**Authors:** Wenjuan Liu, Zhe Su, Junfeng Zhang, Mingjiang Yi, Ling Han, Jingwei Zhou

**Affiliations:** 1 Department of Anesthesiology, Shanghai Tongren Hospital, Shanghai Jiao Tong University School of Medicine, 1111 XianXia Road, Shanghai, 200336 China; 2 Department of Biostatistics, School of Global Public Health, New York University, New York, NY USA

**Keywords:** Ultrasound guidance, Internal jugular vein cannulation, Oblique-axis, Short-axis, Left decubitus position, Randomized controlled trial

## Abstract

**Background:**

Central venous cannulation in the left decubitus position is more difficult than in the supine position and may be required when patients cannot safely return to supine. Whether the oblique-axis (OA) approach improves the success and safety of right internal jugular vein (RIJV) cannulation compared with the short-axis (SA) approach remains unknown.

**Methods:**

Seventy-four patients (ASA I–III) scheduled for elective surgery in the left decubitus position under general anesthesia requiring RIJV cannulation were randomly assigned 1:1 to the OA or SA group (*n* = 37 each). Under real-time ultrasound guidance and a 20° Trendelenburg tilt, cannulation was performed by one of three experienced attending anesthesiologists, each with more than 15 years of experience in ultrasound-guided central venous cannulation. Venipuncture attempts and total procedure time were recorded by a separate investigator; patients were blinded by general anesthesia. The primary outcome was first-time puncture success; secondary outcomes were overall cannulation success, procedure time, venipuncture attempts, and mechanical complications.

**Results:**

All 74 randomized patients completed the trial and were analyzed. The first-time puncture success rate was higher in the OA group (27/37 [73.0%] vs. 18/37 [48.6%]; risk difference 24.3%, 95% CI 2.8% to 45.9%; *P* = 0.032, Pearson chi-square test; Fisher exact test *P* = 0.056). Venipuncture attempts were fewer in the OA group (median 1 vs. 2; IQR 1–2; *P* = 0.037). Total procedure time did not differ significantly between groups (median 2.79 [IQR 2.46–3.19] vs. 3.01 [2.72–3.45] min; *P* = 0.060). Mechanical complications occurred in 6 of 37 patients (16.2%) in the SA group (one arterial puncture and five posterior wall penetrations) and in none in the OA group (*P* = 0.025); overall cannulation success was 100% in both groups.

**Conclusions:**

For RIJV cannulation in the left decubitus position, the oblique-axis approach was associated with a higher first-time puncture success rate than the short-axis approach and with fewer mechanical complications detected during or immediately after the procedure; however, the primary-endpoint evidence was borderline and the complication estimates were imprecise.

**Trial registration:**

Chinese Clinical Trial Registry (ChiCTR2400091385; registered 28 October 2024).

**Supplementary Information:**

The online version contains supplementary material available at https://doi.org/10.1186/s12871-026-04360-3.

## Introduction

Central venous cannulation (CVC) is a commonly performed procedure in emergency, intensive care, and perioperative settings. It provides safe vascular access for the precise administration of potent vasoactive drugs, rapid fluid resuscitation, central venous pressure monitoring, transvenous cardiac pacing, and temporary hemodialysis [1]. The right internal jugular vein (RIJV) is the most commonly used site for perioperative cannulation, with fewer catheter-related infections than the femoral vein [1, 2] and a lower risk of pneumothorax than the subclavian vein [1, 3].

Most studies of CVC have been performed in the supine position. In clinical practice, however, an unplanned need for central venous access occasionally arises in patients who are already positioned for surgery in a non-supine position — for example, semi-recumbent, prone, or lateral decubitus — and repositioning to the supine position is often neither feasible nor safe once the patient is draped and surgically positioned. In the decubitus position, the internal jugular vein (IJV) is frequently the only feasible access site, and the relatively fixed head-and-neck position, the loss of palpable anatomical landmarks, and the increased overlap between the carotid artery and the IJV all increase the technical difficulty of cannulation.

Ultrasound guidance (USG) has fundamentally changed the practice of CVC access. Compared with the landmark technique, USG reduces overall complications, increases first-attempt success rates, and shortens cannulation time [4]. Current guidelines from intensive care [5], anesthesiology [6, 7], and health technology assessment bodies [8], together with international evidence-based recommendations [9] and expert reviews [10–12], recommend real-time USG as the preferred method for central venous access.

The ultrasound-guided oblique-axis (OA) approach is an intermediate orientation between the short-axis (SA) and long-axis views. It allows simultaneous visualization of the puncture needle tip and shaft throughout the procedure while maintaining a side-by-side view of the IJV and carotid artery [13]. Previous studies comparing probe orientations have been performed predominantly in the supine position; the OA approach has rarely been evaluated in special positions such as the left decubitus position. We therefore conducted this prospective, randomized, single-center trial to evaluate the feasibility and safety of the USG OA approach compared with the USG SA approach for RIJV cannulation in the left decubitus position. Because randomized evaluation is not feasible in emergency or critically ill patients who require unplanned central venous access and cannot be repositioned to supine, we studied elective surgical patients scheduled for surgery in the left decubitus position, in whom RIJV cannulation was clinically indicated and the two approaches could be compared under standardized, controlled conditions.

## Methods

### Trial design and ethics

This was a prospective, single-center, parallel-group randomized controlled trial conducted at Shanghai Tongren Hospital, China. This study was performed in accordance with the Declaration of Helsinki and with applicable national regulations governing research involving human participants. The study protocol (version 2.0) was approved by the Ethics Committee of Shanghai Tongren Hospital (No. 2024-067-02) on 17 October 2024, and the trial was registered with the Chinese Clinical Trial Registry (ChiCTR2400091385) on 28 October 2024. Written informed consent was obtained from all participants before enrollment. The CONSORT checklist is provided as Additional file 1. The full trial protocol is available from the corresponding author on reasonable request.

### Participants

Consecutive patients scheduled for elective surgery in the left decubitus position under general anesthesia who required RIJV cannulation were screened during the preoperative interview between 1 November 2024 and 31 December 2025. Inclusion criteria were: (1) age 18–65 years; (2) ASA physical status I–III; (3) body mass index (BMI) 18.5–30 kg/m². Exclusion criteria were: abnormal blood coagulation function; infection at the puncture site; IJV anatomical variations or venous thrombosis detected by ultrasound; superior vena cava syndrome; presence of a central venous catheter in the target vein within the preceding 72 h; and refusal to participate.

### Randomization, allocation concealment, and blinding

Eligible patients were enrolled by the attending anesthesiologist during the preoperative interview and were randomly assigned in a 1:1 ratio to the SA group (control) or the OA group (experimental) using a computer-generated permuted-block randomization sequence with a fixed block size of 6, prepared by an investigator (JW Z) not involved in the procedures. No stratification factors were used. Allocation was concealed in opaque, sealed, sequentially numbered envelopes prepared by an investigator (LH) who was not involved in subsequent procedures; the envelope was opened immediately before surgery by the operating anesthesiologist after confirmation of eligibility. Both the operating anesthesiologists and the recording investigator (MJ Y) were aware of group allocation; the operators could not be blinded because the two techniques require distinct probe orientations and needle trajectories. To reduce—but not eliminate—the risk of bias from unblinded outcome assessment, the number of venipuncture attempts and total procedure time were recorded intraoperatively by MJ Y, who was present throughout the procedure and did not perform the cannulation. Total procedure time was measured with a digital stopwatch that was started and stopped by MJ Y on the operator’s verbal command at the defined start and end points (see Outcomes). Patients were under general anesthesia and therefore unaware of their group assignment. All remaining outcomes, including the diagnosis of mechanical complications, were assessed and recorded in real time by the operating anesthesiologist using the pre-defined criteria.

### Preoperative preparation and positioning

Standard monitoring (electrocardiography, non-invasive blood pressure monitoring, and pulse oximetry) was established, and an 18-G peripheral venous catheter was inserted for infusion of lactated Ringer’s solution. All patients received non-invasive blood pressure monitoring before arterial cannulation. Invasive arterial pressure monitoring was established in all patients before induction of anesthesia because the planned surgery required close intraoperative hemodynamic management, including guidance of fluid therapy and vasoactive drug administration; it was used for these clinical indications rather than as part of routine standard monitoring. Anesthesia was induced with propofol 1–2 mg/kg, sufentanil 0.3–0.4 µg/kg, and rocuronium 0.6 mg/kg, followed by tracheal intubation and mechanical ventilation adjusted to maintain an end-tidal carbon dioxide level of 35–45 mmHg.

After tracheal intubation, patients were placed in a true left lateral decubitus position, with the trunk aligned parallel to the longitudinal axis of the operating table. The head and neck were immobilized in a neutral position with a pillow, avoiding any rotation of the cervical spine. The hips and knees were flexed to nearly 90° to stabilize the position and relax the abdominal wall; the upper leg was kept straight with a soft pad between the legs. Both arms were extended forward, parallel to the torso, and secured with a double brace. To promote venous filling of the RIJV, the surgical bed was tilted to a 20° Trendelenburg position. Before puncture, a linear array probe (6–13 MHz; EDGE II, FUJIFILM Sonosite, USA) was used to scan the RIJV, the carotid artery, and adjacent structures to exclude anatomical variations and venous thrombosis.

### Interventions

All cannulations were performed by one of three attending anesthesiologists (Z S, WJ L, and JF Z) at our center, each with more than 15 years of experience in ultrasound-guided central venous cannulation, using the standard Seldinger technique with an 18-Ga, 6.5-cm introducer needle (Centra-Line™, Biometrix, Israel) attached to a 5-mL syringe. All three operators performed both the oblique-axis (OA) and the short-axis (SA) technique, and the technique used for each patient was determined solely by the randomization sequence. Before the trial, all three operators completed a standardized training program on both approaches and were required to demonstrate proficiency in the oblique-axis technique (each performing a minimum of 20 supervised oblique-axis cannulations) to ensure uniform application of the two techniques across the groups. Each operator managed both the probe and the needle; the probe was enclosed in a sterile cover under standard aseptic conditions. Once the needle tip was identified within the vein, the syringe was removed, a J-wire was advanced, and its position within the IJV was confirmed ultrasonographically. Cannulation was defined as successful if the guidewire advanced smoothly without resistance and was confirmed inside the IJV. After the catheter had been inserted over the guidewire and secured in place, the surgical bed was returned to the horizontal position.

####  SA group (control) 

The ultrasound probe was positioned transversely on the neck, parallel to the clavicle. After clear visualization of the IJV at the center of the ultrasound image, the needle was inserted using an out-of-plane technique, with a trajectory perpendicular to the long axis of the transducer [14]

####  OA group (experimental)

The target vessel was first identified in the standard short-axis orientation. The probe was then rotated to an intermediate position approximately midway between the short-axis and long-axis views [15], maintaining visualization of both the IJV and the carotid artery in a slightly elongated view. The needle was advanced along the long axis of the probe (in-plane), providing real-time visualization of the entire needle trajectory from skin entry to vessel penetration

### Outcomes

The primary outcome was the first-time puncture success rate, defined as successful cannulation achieved with a single venipuncture attempt—that is, venous blood flashback obtained on the first needle pass, followed by smooth guidewire advancement and catheter placement, without any further needle puncture. Under the pre-specified counting rule, in which every successive needle withdrawal and re-advancement (with or without a new skin puncture site) constituted an additional attempt [16], first-time puncture success corresponded to a recorded venipuncture-attempt count of one. Patients with exactly one recorded attempt, as recorded intraoperatively by a designated investigator (MJ Y), were classified as having first-time puncture success.

Secondary outcomes were: (1) overall cannulation success rate — cannulation was classified as failed if the IJV was not cannulated after three venipuncture attempts [17]; (2) total procedure time (min) — the interval from the first needle puncture of the skin to completion of catheter fixation, recorded by MJ Y using a digital stopwatch [17]; the stopwatch was started and stopped by MJ Y on the operator’s verbal command at the defined start and end points, respectively; (3) number of venipuncture attempts — the number of venipuncture attempts required for successful cannulation [17], counted and recorded at the time of the procedure by MJ Y, who was present throughout the procedure and did not perform the cannulation; and (4) mechanical complications — arterial puncture, posterior venous wall penetration, pneumothorax, air embolism, and catheter malposition, detected during or immediately after the procedure. Arterial puncture was defined as needle penetration of the carotid artery [16], which was managed by immediate needle withdrawal and local compression. Posterior venous wall penetration was defined as penetration of the deep (posterior) wall of the IJV by the puncture needle [17], diagnosed by intraoperative ultrasound visualization of the puncture needle traversing the posterior wall of the IJV. Confirmed events were managed by immediate needle withdrawal and re-puncture under real-time ultrasound guidance. All adverse events were managed according to clinical best practice and reported to the ethics committee as required. Posterior venous wall penetration was assessed by the operating anesthesiologist, and the corresponding ultrasound images were not retained. No dedicated imaging or systematic screening protocol was used to detect pneumothorax, air embolism, or catheter malposition; the reported absence of these events reflects that none was detected with the intraoperative monitoring in use and clinical observation during and immediately after the procedure. After surgery, ward staff notified the anesthesiology team only when a problem arose; this was passive reporting without a predefined reporting cutoff, rather than systematic research follow-up. Routine postoperative chest radiographic screening was not performed.

### Sample size

Sample size was calculated on the basis of a pilot study conducted before the main trial, in which 20 patients were randomly assigned in a 1:1 ratio (10 per group) and the first-time puncture success rates were 5/10 (50%) in the experimental group and 2/10 (20%) in the control group. To detect this anticipated 30-percentage-point between-group difference with a two-tailed test for the difference between two proportions at α = 0.05 and β = 0.20 (power 80%), a sample of approximately 37 participants per group was required, giving a total planned sample of 74; all 74 randomized participants completed the trial.

### Statistical analysis

Statistical analyses were performed with SPSS version 21.0 (SPSS Inc., Chicago, IL, USA). Continuous data are presented as mean ± SD or median (interquartile range [IQR]) according to distribution, and categorical data as numbers (percentages). Between-group comparisons of normally distributed continuous variables used the Student’s t test; skewed continuous and ordinal variables (procedure time, number of venipuncture attempts) were compared with the Mann–Whitney U test, For these variables, the between-group location shift is expressed as the Hodges–Lehmann estimate with an approximate 95% confidence interval derived from the ranked pairwise between-group differences (normal approximation with correction for ties); the observed difference between the group medians is reported descriptively without a confidence interval. Categorical variables (first-time puncture success, overall success, and complications) were compared with the chi-square test or Fisher’s exact test, as appropriate. ASA class was reported descriptively and was not subjected to between-group statistical comparison because no patient in the SA group had ASA class I; a dichotomized (I–II vs. III) classification was not used for comparison. The primary outcome analysis used the Pearson chi-square test (pre-specified), with the Fisher exact test as a sensitivity analysis. Between-group differences in binary outcomes are expressed as risk differences with 95% confidence intervals, computed by the Wald method or, when one group had zero events, by the Newcombe hybrid score method; relative risks are reported with 95% confidence intervals computed by the log method. A two-sided *P* < 0.05 was considered statistically significant. No adjustment for multiple testing was made; secondary outcomes were pre-specified but exploratory, and their effect estimates, confidence intervals, and P values are reported without multiplicity correction. All analyses followed the intention-to-treat principle; no interim analysis was planned.

## Results

### Participant flow and baseline characteristics

**Fig. 1:**
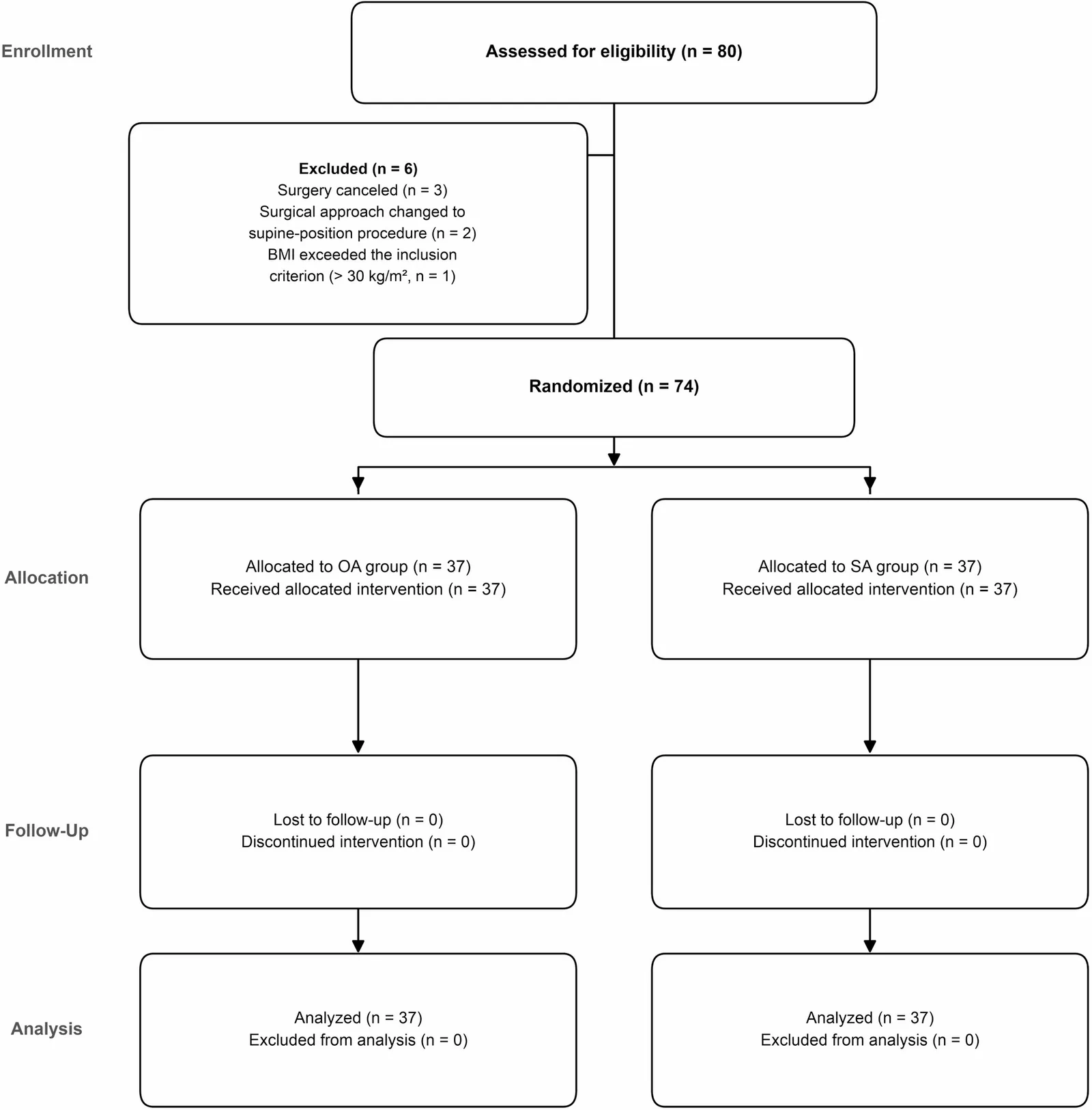
CONSORT flow diagram of participant enrollment, randomization, and analysis. Of 80 patients assessed for eligibility between 1 November 2024 and 31 December 2025, six were excluded before randomization (three because their surgery was canceled, two because the surgical approach was changed to one requiring a supine position, and one because the body mass index exceeded the inclusion criterion), and 74 were randomized (37 per group); all completed the trial

Between 1 November 2024 and 31 December 2025, 80 patients were assessed for eligibility. Six patients were excluded before randomization: three because their surgery was canceled, two because the surgical approach was changed to one requiring a supine position, and one because the body mass index (BMI) exceeded the inclusion criterion (> 30 kg/m²). The remaining 74 patients were randomized, 37 to the OA group and 37 to the SA group. All 74 patients completed the protocol without withdrawal or protocol deviation and were included in the analysis (Fig. 1). Baseline characteristics were similar between the two groups (Table 1).

**Table 1:** Baseline characteristics of the study participants

Variable	OA group (*n* = 37)	SA group (*n* = 37)
Age (years), mean ± SD	49.2 ± 15.6	50.5 ± 14.4
Male, n (%)	19 (51.4)	21 (56.8)
Height (cm), mean ± SD	167.0 ± 9.3	164.5 ± 7.6
Weight (kg), mean ± SD	68.1 ± 13.1	65.6 ± 14.1
BMI (kg/m²), mean ± SD	24.3 ± 3.5	24.1 ± 4.3
ASA class I, n (%)	2 (5.4)	0 (0)
ASA class II, n (%)	8 (21.6)	9 (24.3)
ASA class III, n (%)	27 (73.0)	28 (75.7)

Continuous variables are presented as mean ± SD; categorical variables as n (%)

OA, oblique-axis; SA, short-axis; SD, standard deviation; BMI, body mass index; ASA, American Society of Anesthesiologists

### Primary outcome

The first-time puncture success rate was significantly higher in the OA group than in the SA group (27/37 [73.0%] vs. 18/37 [48.6%]; risk difference 24.3%, 95% CI 2.8% to 45.9%; RR 1.50, 95% CI 1.02 to 2.20; *P* = 0.032, Pearson chi-square test). The pre-specified Fisher exact sensitivity analysis did not meet the two-sided 0.05 significance threshold (*P* = 0.056).

### Secondary outcomes

**Fig. 2:**
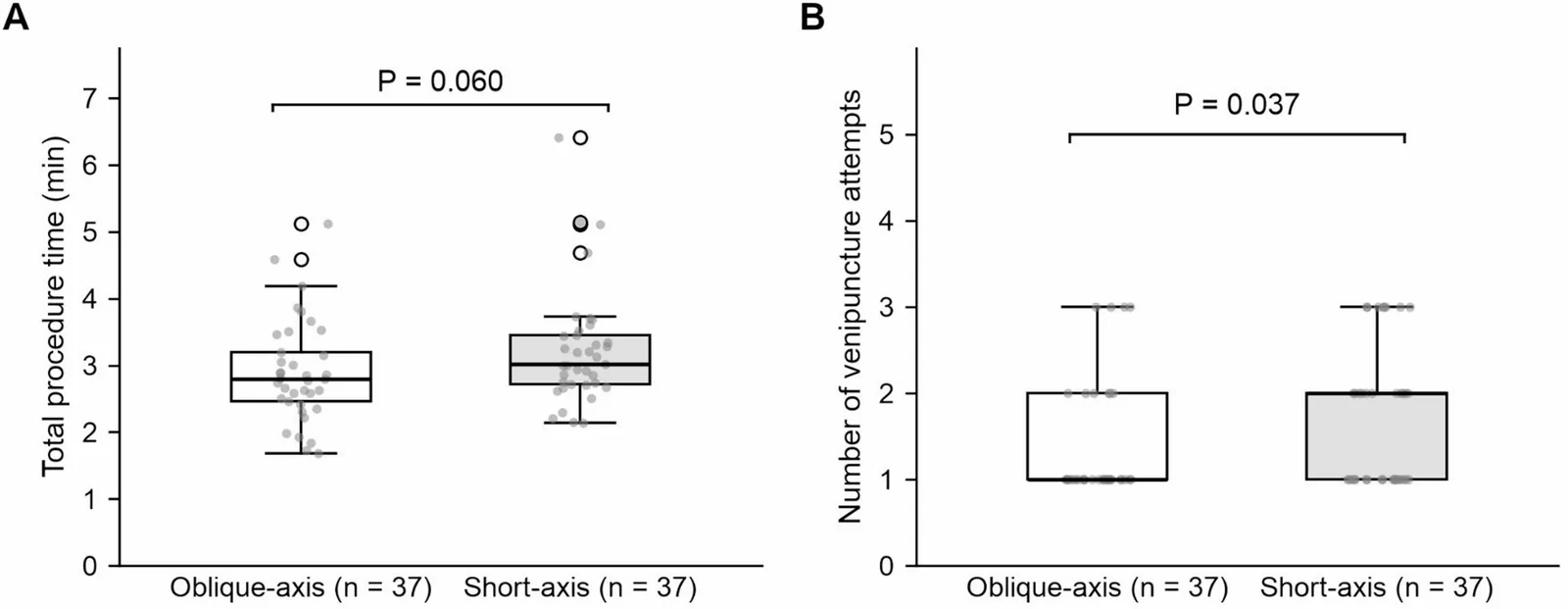
Box-and-whisker plots of (A) total procedure time and (B) number of venipuncture attempts by group

The number of venipuncture attempts was significantly lower in the OA group than in the SA group (median 1 vs. 2; IQR 1–2 for both groups; Hodges–Lehmann estimate [SA − OA] 0 attempts, 95% CI 0 to 1; observed difference between group medians 1 attempt; *P* = 0.037, Mann–Whitney U test; Fig. 2). Total procedure time tended to be shorter in the OA group (2.90 ± 0.76 min; median 2.79, IQR 2.46–3.19) than in the SA group (3.24 ± 0.88 min; median 3.01, IQR 2.72–3.45), but the difference did not reach statistical significance (Hodges–Lehmann estimate [SA − OA] 0.28 min, 95% CI − 0.01 to 0.59; *P* = 0.060, Mann–Whitney U test). The overall cannulation success rate was 100% in both groups (37/37 vs. 37/37).

Mechanical complications occurred in 6 of 37 patients (16.2%) in the SA group — one arterial puncture and five posterior venous wall penetrations, each in a different patient — and in none of the 37 patients in the OA group (risk difference − 16.2%, 95% CI − 31.1% to − 3.5%; *P* = 0.025, Fisher exact test; Table 2). No pneumothorax, air embolism, or catheter malposition was detected during or immediately after the procedure in either group. All detected complications were managed as described in the Methods.

**Table 2:** Study outcomes

Outcome	OA group (*n* = 37)	SA group (*n* = 37)	Effect (95% CI)	*P* value
Primary outcome				
First-time puncture success, n (%)	27 (73.0)	18 (48.6)	RD 24.3% (2.8% to 45.9%); RR 1.50 (1.02 to 2.20)	0.032*
**Secondary outcomes**				
Overall cannulation success, n (%)	37 (100)	37 (100)	—	—
Total procedure time (min), mean ± SD	2.90 ± 0.76	3.24 ± 0.88	—	—
Total procedure time (min), median (IQR)	2.79 (2.46–3.19)	3.01 (2.72–3.45)	Hodges–Lehmann estimate (SA − OA) 0.28 min (95% CI − 0.01 to 0.59)¶	0.060‡
Venipuncture attempts, median (IQR)	1 (1–2)	2 (1–2)	Hodges–Lehmann estimate (SA − OA) 0 attempts (95% CI 0 to 1)¶; observed median difference 1 attempt	0.037‡
Mechanical complications, n (%)				
Arterial puncture	0 (0)	1 (2.7)	—	1.000
Posterior venous wall penetration	0 (0)	5 (13.5)	—	0.054
Pneumothorax	0 (0)	0 (0)	—	—
Air embolism	0 (0)	0 (0)	—	—
Catheter malposition	0 (0)	0 (0)	—	—
Any mechanical complication	0 (0)	6 (16.2)	RD − 16.2% (− 31.1% to − 3.5%)§	0.025

OA, oblique-axis; SA, short-axis; RD, risk difference; RR, relative risk; IQR, interquartile range; CI, confidence interval

* Pearson chi-square test (pre-specified primary analysis); Fisher exact test sensitivity analysis *P* = 0.056

‡ Mann–Whitney U test

§ Risk difference with 95% CI computed by the Newcombe hybrid score method (the OA group had zero events)

¶ Approximate 95% CI obtained by the Hodges–Lehmann method (normal approximation with correction for ties, using ranked pairwise between-group differences). With these coarse, heavily tied data, this method gives a Hodges–Lehmann location-shift estimate of 0 attempts with a 95% CI of 0 to 1 attempts; the observed difference between the group medians is 1 attempt, reported descriptively without a confidence interval

Secondary outcomes were pre-specified but exploratory; no adjustment for multiple testing was made. —, not estimable (100% success or no events in both groups) or not reported (sparse individual complications). All mechanical complications reported in Table 2 were those detected during or immediately after the procedure

## Discussion

In this randomized controlled trial of ultrasound-guided RIJV cannulation in the left decubitus position, the oblique-axis approach achieved a higher first-time puncture success rate (73.0% vs. 48.6%) and was associated with fewer mechanical complications (0% vs. 16.2%) than the short-axis approach. These findings provide preliminary evidence of a potential technical benefit of the OA approach in this position, but do not establish a definitive safety advantage or address delayed catheter-related complications. Central venous cannulation remains one of the most commonly performed procedures in perioperative and critical care practice, with millions of central venous catheters inserted each year [18], and is associated with substantial catheter-related morbidity [3]. Real-time ultrasound guidance has become the standard of care for central venous access, and its benefit is greatest when the needle is tracked in-plane during vessel puncture [19].

The SA approach is traditionally preferred by clinicians for performing and teaching ultrasound-guided IJV cannulation, but it is associated with a higher rate of posterior venous wall penetration because the needle is visualized only as a cross-sectional dot and its depth within the vein cannot be reliably tracked [14, 16, 17]. In the decubitus position, gravitational effects collapse the IJV and reduce its cross-sectional area, further increasing the technical challenge [20, 21]. Consistent with this, in the present study all five posterior wall penetrations and the single arterial puncture occurred in the SA group. These events were diagnosed in real time by the unblinded operating anesthesiologist. The oblique-axis orientation offers the advantages of both the short- and long-axis views: the needle is followed in real time from skin to vein while the IJV and carotid artery remain visible side by side, which should reduce both posterior wall penetration and inadvertent arterial injury [13, 22]. The number of venipuncture attempts and the rate of posterior wall penetration in the SA group in our study are consistent with those reported in previous comparisons of probe orientations [14, 17, 23, 24].

The observed reduction in posterior wall penetration (13.5% vs. 0%) may be clinically relevant: posterior wall penetration can produce hematoma and, in the setting of increased arteriovenous overlap, increases the probability of arterial injury [20]. The higher number of venipuncture attempts in the SA group (median 2 vs. 1) may itself be a contributing mechanism, because repeated puncture increases the cumulative risk of posterior wall penetration; conversely, in-plane visualization of the full needle shaft may have reduced the need for redirection in the OA group. Because the between-group comparison of mechanical complications rests on only six events in total, this estimate is correspondingly fragile and its confidence interval wide (risk difference − 16.2%, 95% CI − 31.1% to − 3.5%); it should therefore be regarded as preliminary and requires confirmation in larger samples.

The interpretation of the primary endpoint deserves particular emphasis. First-time puncture success occurred in 27/37 patients in the OA group and 18/37 in the SA group. With rows defined as OA and SA and columns as success and failure, the expected cell counts were 22.5 and 14.5 in each row; all exceeded five, supporting use of the pre-specified Pearson chi-square approximation. The primary analysis met the two-sided 0.05 significance threshold (*P* = 0.032), whereas the Fisher exact sensitivity analysis (*P* = 0.056) and the chi-square test with Yates’ continuity correction (*P* = 0.057) did not. Fisher’s exact test conditions on the margins and can be conservative because its attainable P values are discrete; nevertheless, its result should not be dismissed on that basis. It serves as a sensitivity analysis rather than a replacement for the pre-specified primary analysis, and the difference between tests indicates that threshold-based inference is not robust. The risk difference was 24.3% points (95% CI 2.8 to 45.9), and the relative risk was 1.50 (95% CI 1.02 to 2.20). These estimates favor OA, but their confidence intervals are wide. They are alternative summaries of the same outcome data, not independent replications, and the Wald risk-difference interval is not obtained by inverting Fisher’s exact test; its exclusion of zero therefore does not resolve the test-dependent inference. Reclassifying a single outcome would move the Pearson chi-square result across the 0.05 threshold, illustrating the sensitivity of the significance conclusion. The anticipated 30-percentage-point difference used for sample-size planning came from a pilot study of only 20 patients (5/10 vs. 2/10), with a very imprecise estimated risk difference (95% CI approximately − 10 to 70% points); this assumption may have been optimistic relative to the smaller difference observed in the main trial. The mechanical-complication result is a separate exploratory outcome based on only six events, not confirmatory evidence of superiority. Likewise, the lower number of venipuncture attempts in the OA group (*P* = 0.037, Mann–Whitney U test) is not independent corroboration, because the primary outcome was derived from the same recorded attempt count. Together with unblinded outcome assessment, these limitations warrant larger confirmatory trials before firm recommendations are made.

Several additional points merit discussion. First, the point estimates for total procedure time favored OA, but the between-group difference did not reach statistical significance (*P* = 0.060; Hodges–Lehmann estimate [SA − OA] 0.28 min, 95% CI − 0.01 to 0.59). This is not evidence of equivalence or of a demonstrated time advantage. The interval began at the first skin puncture and therefore excluded pre-puncture ultrasound scanning and probe repositioning, including rotation to obtain the oblique-axis view. Whether including these preparatory steps would increase or decrease the between-group difference is unknown. Future studies should record the access interval—from the first skin puncture to smooth guidewire advancement with ultrasound confirmation of intravascular position—separately from the overall procedural interval. Second, all three operators had more than 15 years of ultrasound-guided central venous cannulation experience and performed both approaches after standardized pre-trial training, including at least 20 supervised OA cannulations per operator. This defines the training context in which the findings were obtained; it does not establish a learning curve, validate 20 procedures as a competence threshold, or demonstrate generalizability to trainees. Future studies should record operator-specific caseloads and outcomes and assess performance across experience levels.

### Limitations

This study has several limitations. First, it was a single-center trial with a modest sample size (*n* = 74). The sample size was planned for a 30-percentage-point difference in first-time puncture success using a small pilot study (5/10 vs. 2/10), whereas the observed difference was smaller, at 24.3% points; the pilot estimate was imprecise, so the adequacy of the planned sample size for detecting smaller effects remains uncertain. The modest sample size also limits the precision of the secondary-outcome and complication estimates and restricts the scope for reliable operator-specific analyses and multivariable adjustment. Second, inclusion was restricted to adults aged 18–65 years with ASA I–III and BMI 18.5–30 kg/m²; whether the findings extend to older, higher-ASA, or obese patients requires further study. Third, only elective patients undergoing right-sided RIJV cannulation in the left decubitus position were studied, and all cannulations were performed by three attending anesthesiologists with more than 15 years of experience after standardized training that included at least 20 supervised oblique-axis cannulations each. Although each operator performed both techniques, this does not establish equivalent operator allocation between groups, and the findings should therefore be interpreted with the possibility of an operator-by-technique interaction in mind. They should also not be extrapolated to unplanned cannulation in emergency or critically ill patients, to trainees or less experienced practitioners, or to centres without established expertise in the oblique-axis view. Fourth, blinding of the operators was not feasible because the two techniques require distinct probe orientations and needle trajectories. The number of venipuncture attempts and the total procedure time were recorded by a separate, unblinded investigator (MJ Y), but the diagnosis of mechanical complications was made by the unblinded operating anesthesiologist without independent adjudication, and the corresponding ultrasound images were not retained. Expectation bias and differential misclassification of these operator-assessed outcomes therefore cannot be excluded; the direction and magnitude of any resulting bias cannot be determined from these data. Fifth, safety ascertainment was restricted to mechanical complications detected during or immediately after the procedure, with no dedicated post-cannulation surveillance for hematoma progression, catheter-related thrombosis, or catheter-related bloodstream infection; delayed sequelae, including those after the five posterior venous wall penetrations, were not systematically ascertained. Postoperative information relied on problem-triggered reports from ward staff to the anesthesiology team, without a predefined reporting cutoff or routine chest radiographic screening. Routine postoperative clinical care should not be equated with protocol-defined research follow-up, and the absence of a reported late event must not be interpreted as evidence that none occurred; no conclusion about delayed catheter-related safety can therefore be drawn, and future studies require predefined surveillance windows and outcome definitions. Sixth, total procedure time was defined as the interval from the first skin puncture to completion of catheter fixation and therefore excluded pre-puncture ultrasound scanning and probe repositioning while including guidewire advancement, dilation, catheter insertion, and fixation. Although both groups followed the same standardized Seldinger protocol, the direction of any resulting bias cannot be determined from these data. Seventh, neck circumference, skin-to-IJV depth, and the degree of truncal rotation were not recorded; ultrasound screening excluded anatomical variations and venous thrombosis but does not provide quantitative anatomical characterization, and BMI is not a substitute for direct measurement of vessel depth or neck anatomy. Randomization would be expected to balance such unmeasured characteristics on average; however, chance imbalance between the two groups in this sample cannot be excluded, and because these variables were not measured, an anatomy-by-technique interaction also cannot be excluded. Future studies should prospectively record standardized anatomical measures.

## Conclusions

In patients undergoing RIJV cannulation in the left decubitus position under general anesthesia, the ultrasound-guided oblique-axis approach was associated with a higher first-time puncture success rate than the short-axis approach and with fewer mechanical complications detected during or immediately after the procedure; however, the primary-endpoint finding was borderline and sensitive to the choice of statistical test. These findings suggest a potential technical benefit of the OA approach for ultrasound-guided IJV cannulation in the decubitus position, but they should be regarded as preliminary, and larger multicenter trials are needed to confirm them before any recommendation can be made.

## Supplementary Information

Below is the link to the electronic supplementary material.

Supplementary Material 1

## Data Availability

Availability of data and materials: The datasets analyzed during the current study are available from the corresponding author on reasonable request.

## Abbreviations

ASA: American Society of Anesthesiologists
BMI: body mass index
CI: confidence interval
CVC: central venous cannulation
IJV: internal jugular vein
IQR: interquartile range
OA: oblique-axis
RD: risk difference
RIJV: right internal jugular vein
RR: relative risk
SA: short-axis
USG: ultrasound guidance
