# Supplementary material for: Comparison of ultrasound-guided oblique-axis versus short-axis approaches for right internal jugular vein cannulation in the left decubitus position: a prospective, randomized, single-center trial
Source: BMC Anesthesiol. 2026 Oct 19;26:651. doi: 10.1186/s12871-026-04360-3 (PMC13726049; doi:10.1186/s12871-026-04360-3)
Supplement: Supplementary file 1 [file 12871_2026_4360_MOESM1_ESM.docx]

**CONSORT 2010 checklist of information to include when reporting a randomised trial***

Manuscript: Comparison of ultrasound-guided oblique-axis versus short-axis approaches for right internal jugular vein cannulation in the left decubitus position: a prospective, randomized, single-center trial (submitted to BMC Anesthesiology)

Completed against the submitted manuscript OAvsSA_manuscript(BMCA).docx, 6 September 2026.

| **Section/Topic** | **Item No** | **Checklist item** | **Reported on page No.** |
| --- | --- | --- | --- |
| **Title and abstract** | | | |
| Title and abstract | 1a | Identification as a randomised trial in the title | 1 |
| Title and abstract | 1b | Structured summary of trial design, methods, results, and conclusions (for specific guidance see CONSORT for abstracts) | 1-2 |
| **Introduction** | | | |
| Introduction | 2a | Scientific background and explanation of rationale | 2 |
| Introduction | 2b | Specific objectives or hypotheses | 2 |
| **Methods** | | | |
| Methods | 3a | Description of trial design (such as parallel, factorial) including allocation ratio | 3 |
| Methods | 3b | Important changes to methods after trial commencement (such as eligibility criteria), with reasons | None |
| Methods | 4a | Eligibility criteria for participants | 3 |
| Methods | 4b | Settings and locations where the data were collected | 3 |
| Methods | 5 | The interventions for each group with sufficient details to allow replication, including how and when they were actually administered | 4 |
| Methods | 6a | Completely defined pre-specified primary and secondary outcome measures, including how and when they were assessed | 4 |
| Methods | 6b | Any changes to trial outcomes after the trial commenced, with reasons | None |
| Methods | 7a | How sample size was determined | 6 |
| Methods | 7b | When applicable, explanation of any interim analyses and stopping guidelines | 6 |
| Methods | 8a | Method used to generate the random allocation sequence | 3 |
| Methods | 8b | Type of randomisation; details of any restriction (such as blocking and block size) | 3 |
| Methods | 9 | Mechanism used to implement the random allocation sequence (such as sequentially numbered containers), describing any steps taken to conceal the sequence until interventions were assigned | 3 |
| Methods | 10 | Who generated the random allocation sequence, who enrolled participants, and who assigned participants to interventions | 3 |
| Methods | 11a | If done, who was blinded after assignment to interventions (for example, participants, care providers, those assessing outcomes) and how | 3、11 |
| Methods | 11b | If relevant, description of the similarity of interventions | Not applicable |
| Methods | 12a | Statistical methods used to compare groups for primary and secondary outcomes | 6 |
| Methods | 12b | Methods for additional analyses, such as subgroup analyses and adjusted analyses | 3 |
| **Results** | | | |
| Results | 13a | For each group, the numbers of participants who were randomly assigned, received intended treatment, and were analysed for the primary outcome | 7 |
| Results | 13b | For each group, losses and exclusions after randomisation, together with reasons | 7 |
| Results | 14a | Dates defining the periods of recruitment and follow-up | 7 |
| Results | 14b | Why the trial ended or was stopped | 7 |
| Results | 15 | A table showing baseline demographic and clinical characteristics for each group | 13 |
| Results | 16 | For each group, number of participants (denominator) included in each analysis and whether the analysis was by original assigned groups | 7 |
| Results | 17a | For each primary and secondary outcome, results for each group, and the estimated effect size and its precision (such as 95% confidence interval) | 8 |
| Results | 17b | For binary outcomes, presentation of both absolute and relative effect sizes is recommended | 8 |
| Results | 18 | Results of any other analyses performed, including subgroup analyses and adjusted analyses, distinguishing pre-specified from exploratory | None |
| Results | 19 | All important harms or unintended effects in each group (for specific guidance see CONSORT for harms) | 8 |
| **Discussion** | | | |
| Discussion | 20 | Trial limitations, addressing sources of potential bias, imprecision, and, if relevant, the multiplicity of analyses | 10 |
| Discussion | 21 | Generalizability (external validity, applicability) of the trial findings | 9-11 |
| Discussion | 22 | Interpretation consistent with results, balancing benefits and harms, and considering other relevant evidence | 9 |
| **Other information** | | | |
| Other information | 23 | Registration number and name of trial registry | 2 |
| Other information | 24 | Where the full trial protocol can be accessed, if available | 3 |
| Other information | 25 | Sources of funding and other support (such as the supply of drugs), the role of funders | 12 |

*Citation: Schulz KF, Altman DG, Moher D, for the CONSORT Group. CONSORT 2010 Statement: updated guidelines for reporting parallel group randomised trials. BMC Medicine. 2010;8:18.
